# *Eperua oleifera* Ducke (Fabaceae) Oilresin Chemical Composition and the Isolation of a Natural Diterpenic Acid Methyl Ester

**DOI:** 10.1002/cbdv.202501730

**Published:** 2025-09-25

**Authors:** Rayssa Ribeiro, Henrique M. G. Pereira, Alvicler Magalhães, Fernando Hallwass, Monica C. Padilha, Valdir F. Veiga‐Junior

**Affiliations:** ^1^ Department of Chemical Engineering Military Institute of Engineering Rio De Janeiro Brazil; ^2^ Chemistry Institute, Brazilian Doping Control Laboratory (LBCD/IQ–UFRJ) Federal University of Rio de Janeiro Rio de Janeiro Brazil; ^3^ Chemistry Institute (IQ–UFRJ) Federal University of Rio de Janeiro Rio de Janeiro Brazil

**Keywords:** diterpene acids, *Eperua oleifera* Ducke, GC–MS, hardwickiic acid methyl ester, HRMS

## Abstract

*Eperua oleifera* Ducke, commonly known as “copaíba‐jacaré,” produces oilresins traditionally used in folk medicine for wound healing and as antifungal and antibacterial agents, similar to those of *Copaifera* species. However, its chemical composition remains poorly characterized. This study investigated the oilresin of *E. oleifera* collected in the Brazilian Amazon region. After derivatization, the sample was analyzed by GC–MS, with data compared to literature, mass spectral libraries, and standards previously isolated. In addition, neutral and acidic fractions were separated using ion‐exchange open‐column chromatography with silica modified with KOH‐impregnated silica as the stationary phase. Twelve diterpenes were identified, including nine carboxylic acids and three alcohols. Hardwickiic acid and copalic acid were the major components, fully characterized by NMR. The most abundant compound in the neutral fraction was isolated and identified by HRMS and NMR as the methyl ester of hardwickiic acid—a compound not previously reported in oilresins. The application of GC–MS enabled the identification of diterpenes based on known fragmentation patterns. In addition, the direct infusion experiment detected a methyl ester that, to our knowledge, has not been previously reported in oilresins.

## Graphical abstract

Eperua oleifera (Fabaceae) oilresin presents not only diterpenic acids, but also diterpenic acid methyl ester.

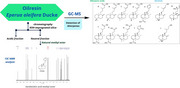

## 1 Introduction

Oilresins are very peculiar vegetal exudates with singular terpenic natural products that defy the common sense of extraction and composition of plant chemodiversity. They are composed only of terpenes and are constituted by mixtures of terpenoids from several classes. They are usually formed by the volatile liquid terpenoids, monoterpenes and sesquiterpenes, that solubilize resinous heavy terpenes, diterpenes and triterpenes. For example, *Pinus* oilresins are composed of aromatic diterpenic acids and monoterpenes. Incense and Mirha from *Olibanum* and *Boswellia* genera, and also *Protium* species, all of them from the Burseraceae family, are composed of monoterpenes and triterpenes, mainly from lupane and ursane backbones. *Copaifera* oilresins (Fabaceae), or copaiba oils, are composed of liquid volatile sesquiterpenes and resinoid diterpenes. They are also produced distinctively and stored in large, primarily schizogenous channels inside the tree trunk. Thus, oilresins are not extracted but naturally exuded from tree trunks. To obtain the pure oilresin, only filtration is necessary to remove parts of the trunk. No solvent or pressure is used. Commercially, they can be produced by cutting the trunk (*Pinus*) or drilling holes in it (*Copaifera*), and slowly flow from the tree [1, 2, 3].

The oilresins found in the Amazon region primarily come from the genera *Protium* (Burseraceae)*, Copaifera*, and *Eperua* (Fabaceae). These oilresins are widely known and used in traditional medicine for their wound‐healing, anti‐inflammatory, and analgesic properties and for treating ulcers. In addition, *Protium* species (commonly known as Breu Branco) are traditionally used as natural insect repellents in the form of incense, for caulking boats, and especially as fragrance fixatives. Copaiba oil is used in the cosmetics industry to produce soaps, perfumes, shampoos, conditioners, and as a renewable fuel source [4, 5, 6]. However, despite their widespread use, one of the main issues in the popular commercialization of medicinal plants is the misidentification of species (by vendors or suppliers) due to morphological similarities. As a result, different species are often sold under the same or similar names and for similar purposes. For example, *Eperua oleifera* is commonly called *copaíba‐jacaré* [7], a name that resembles the yellow copaiba oils, but with an alligator (in Portuguese: *jacaré*) colour, since they are greenish‐dark resins.

*Eperua* Aublet is a genus in the Fabaceae botanical family. Taxonomically, it is very similar to *Copaifera*, with 14 species described to date, predominantly distributed from the Amazon region to Central America, where the trees can reach up to 70 m in height [8, 9]*. Eperua* species produce oilresins that are very similar to those of *Copaifera*. Chemically, *Eperua* oilresins are composed primarily of diterpenes with clerodane and labdane skeletons, closely resembling those found in *Copaifera*. They are also mainly mono and dicarboxylic acids, sometimes minor alcohols. However, *Eperua* oilresins are unique among known oilresins in that they contain exclusively diterpenes, resulting in a much more viscous material [5, 10, 11, 12]. Phytochemical studies have described the oleoresins of *Eperua purpurea* and *Eperua leucantha*, which exhibit high chemical similarity and consist primarily of diterpenic acids and minor alcohols. The main diterpenes identified in these species belong to the labdane‐type structural class, including copalic, cativic, eperuic acids, and 8(17)‐labden‐15‐yl 8(17)‐labden‐15‐oate [13, 14, 15]. Moreover, a cytotoxicity study against tumor cell lines using the oilresin of *E. oleifera* Ducke identified copalic acid and hardwickiic acid as major constituents [12, 16]. However, despite its pharmacological potential, few studies have been conducted on *E. oleifera* to confirm its bioactivity in relation to its chemical composition. In the present study, various analytical strategies were employed to characterize the oleoresin of *E. oleifera* Ducke, collected in the Amazon region. The sample was analyzed by gas chromatography–mass spectrometry (GC–MS) after chemical derivatization, using several standards previously isolated by our research group. Ultimately, a naturally occurring methyl ester was isolated, and its structure was confirmed by nuclear magnetic resonance (NMR) spectroscopy, highlighting the significance of this finding, as esters had not previously been reported in oleoresins.

## 2 Results and Discussion

### 2.1 Phytochemical Analysis

**FIGURE 1:**
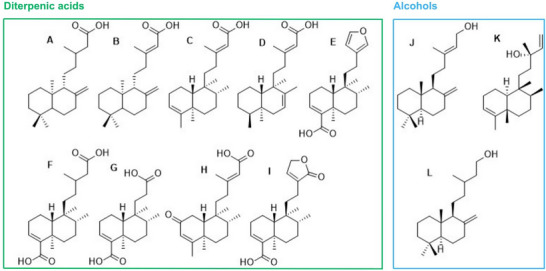
Substances detected as their methyl esters by GC–MS: eperuic acid (A), copalic acid (B), kolavenic acid (C), cleroda‐7,13*E*‐dien‐15‐oic acid (D), hardwickiic acid (E), clerod‐3‐en‐15,18‐dioic acid (F), 14,15,16‐trinor‐hardwickiic acid (G), 2‐oxokolavenic acid (H), patagonic acid (I), labda‐8(20),13*E*‐dien‐15‐ol (J), kolavelool (K), and labd‐8(20)‐en‐15‐ol (L).

GC analysis of the derivatized crude oil revealed the presence of 30 compounds (Figure S1). These were identified using the National Institute of Standards and Technology (NIST) reference libraries and literature data based on MS fragmentation patterns of previously characterized compounds. Among these 30 compounds, 12 showed a match above 90% with the NIST database and were further confirmed through bibliographic references (Figure 1). The detected compounds belong to the terpenoid class, predominantly diterpenic acids, which have been previously described in copaiba oilresin and other *Eperua* species. This finding supports the traditional therapeutic use of *E. oleifera* oilresin for similar medicinal purposes to *Copaifera* oilresins. Indeed, the typical gas chromatographic systems used in natural products research often employ low amounts of phenyl in methyl‐silicone as stationary internal phases, such as DB‐5 or SE‐5, which do not provide good resolution for carboxylic acids. Therefore, derivatization to convert these groups into less polar and better resolved methyl esters has always been state‐of‐the‐art. Consequently, the detected substances included carboxylic acids (identified as their respective methyl esters) and alcohols.

Copalic and hardwickiic acids (detected as their methyl esters) were the major constituents, with relative abundances of 16.0% and 62.8%, respectively. These compounds were confirmed by their characteristic fragmentation patterns and base ion values: *m*/*z* 114 for methyl copalate and *m*/*z* 139 for methyl hardwickiate [17]. Copalic acid is widely reported in the literature, particularly as the only diterpene consistently found across all copaiba oilresins. Consequently, several pharmacological studies have documented its anti‐inflammatory, antibacterial, antifungal, antiparasitic, and cytotoxic activities against tumor cell lines [18]. Similar to copalic acid, hardwickiic acid exhibits pharmacological properties, such as anti‐inflammatory, antibacterial, antiparasitic, antifungal, and antitumor activities [19, 20, 21, 22]. However, despite being reported in numerous phytochemical studies, the biological evaluation of isolated hardwickiic acid remains challenging due to its low concentration in natural sources.

Eperuic acid, named as such because it was first described in *Eperua* species, was also identified based on the fragmentation patterns of the derivatized compound. In addition, other diterpenic acids, including 14,15,16‐trinor‐hardwickiic acid and 2‐oxokolavenic acid, previously reported as constituents in the seeds of *E. leucantha*, as well as clerod‐7,13*E*‐dien‐15‐oic acid, described in *E. purpurea*, were also identified [23, 24]. Another class of diterpenes detected included the alcohols labd‐8(17),13*E*‐dien‐15‐ol and labd‐8(20)‐en‐15‐ol, both known as chemical constituents of *E. purpurea* [13, 14]. Furthermore, additional diterpenes not previously reported in *Eperua* species were identified, following phytochemical data from *Copaifera* species. These include kolavenic acid, clerod‐3‐en‐15,18‐dioic acid, and patagonic acid. Kolavelool was previously characterized in the species *Hardwickia pinnata* [17, 25] (Table 1). The mass spectra were preliminarily compared with the electronic database NIST and then confirmed with all the reference data from the literature [17].

**TABLE 1:** Alcohols and diterpene acids detected as their methyl esters by GC–MS and their corresponding parameters.

Compound	Molecular formula [MF]	Retention time (min)	Base peak (*m*/*z*)	Fragmentation (GC) EIMS *m*/*z*
Labd‐8(20)‐en‐15‐ol	C_22_H_38_O_2_	15.845	137.20	292 (M+, 10%), 277 (25), 177 (40), 149 (8), 137 (100), 123 (30), 109 (32), 95 (70), 81 (70)
Eperuic acid methyl ester	C_21_H_36_O_2_	16.304	137.20	320 (M+, 20%), 305 (60), 289 (5), 277 (7), 249 (7), 223 (10), 203 (7), 191 (10), 177 (62), 149 (15), 137 (100), 123 (35), 109 (45), 95 (90), 81 (85)
Labd‐8(20),13*E*‐dien‐15‐ol	C_20_H_34_O	16.801	81.10	290 (M, 6%), 275 (18), 257 (35), 244 (7), 229 (10), 189 (10), 177 (15), 161 (20), 149 (25) 137 (57), 123 (40), 109 (50), 95 (90), 81 (100), 69 (75), 55 (60), 41 (70)
Copalic acid methyl ester	C_21_H_34_O_2_	19.125	114.10	318 (M+, 5%), 303 (35), 286 (3), 271 (10), 244 (15), 205 (18), 189 (10), 177 (20), 161 (8), 149 (25), 137 (75), 114 (100), 109 (55), 95 (85), 81 (90), 69 (65), 55 (50), 41 (52)
14,15,16‐Trinor‐hardwickiic acid methyl ester	C_19_H_30_O_2_	19.286	290.20	322 (M+, 3%), 290 (100), 275 (5), 235 (10), 203 (20), 175 (28), 139 (32), 107 (20), 91(20)
Kolavenic acid methyl ester	C_21_H_34_O	19.751	189.15	318 (M+, 5%), 303 (10), 275 (8), 271 (10), 243 (20), 235 (18), 191 (32), 189 (100), 175 (32), 161 (18), 149 (22), 135 (42), 121 (68), 107 (100), 95 (95), 81 (40), 67 (35), 55 (45)
Cleroda‐7,13*E*‐dien‐15‐oic acid	C_20_H_32_O_2_	20.715	95.10	318 (M+, 5%), 303 (8), 287 (8), 275 (8), 271 (10), 241 (20), 189 (98), 175 (20), 161 (18), 147 (22), 135 (30), 120 (60), 107 (82), 95 (100), 81 (40), 69 (30), 55 (50)
Hardwickiic acid methyl ester	C_21_H_30_O_3_	23.544	139.15	330 (M+, 8%), 315 (5), 299 (8), 283 (12), 235 (40), 219 (5), 203 (58), 175 (10), 151 (22), 139 (100), 119 (18), 107 (30), 96 (63), 81 (52)
Clerod‐3‐en‐15,18‐dioic acid dimethyl ester	C_29_H_48_O_3_	26.775	332.25	333 (M+, 20%), 332 (100), 235 (30), 219 (8), 203 (50), 175 (30), 151 (22), 139 (90) 119 (20), 107 (30), 91 (20), 79 (20), 55 (22)
2‐Oxokolavenic acid methyl ester	C_15_H_26_O	28.952	95.10	332 (M+, 3%), 285 (5),135 (10), 121 (38), 105 (10), 95 (100), 81 (50)
Kolavelool	C_20_H_34_O	29.991	95.10	290 (M+, 2%), 287 (18), 233 (10), 219 (10), 201 (12), 189 (30), 173 (22), 163 (20), 149 (20), 135 (25), 121 (42), 107 (42), 95 (100), 81 (95), 67 (28), 55 (42)
Patagonic acid methyl ester	C_21_H_30_O_2_	37.888	314.20	346 (M+, 2%), 315 (22%), 314 (100), 299 (7), 271 (10), 203 (8), 175 (30), 139 (20), 119 (15), 105 (25), 91 (30), 79 (15), 67 (10), 55 (20)

Some of the detected diterpenes are rare substances that have not been previously studied biologically. Sometimes, only in mixtures, extracts, or even oilresins. For instance, labda‐8(17),13*E*‐dien‐15‐ol and kolavenic acid have demonstrated antibacterial properties [26, 27]. Moreover, kolavenic and 2‐oxokolavenic acids have been reported for their evident antifungal activities [28]. These findings support the rationale for the therapeutic use of the oilresin from *E. oleifera* for such purposes.

Thanks to the extensive research using this tool for terpene analysis, the GC–MS technique allowed the identification of the main compounds without the need for isolation. This study enhances our understanding of *E. oleifera* oilresin and its constituents, directing future research on isolation, biological activity testing, and more sensitive identification techniques.

The hybrid quadrupole‐orbitrap mass spectrometer offers a range of scan modes that provide functionality comparable to that of conventional tandem quadrupole mass spectrometers. As examples, the product ion, fragment ion, and data‐dependent neutral loss trigger scan modes. Most importantly, the Orbitrap analyzer of the Q Exactive mass spectrometer is a trapping device, not a scanning device [29]. This tool offers greater sensitivity compared to analytical methods commonly used in the analysis of natural products, which will be further applied to *Eperua* oilresins.

### 2.2 Isolation

**FIGURE 2:**
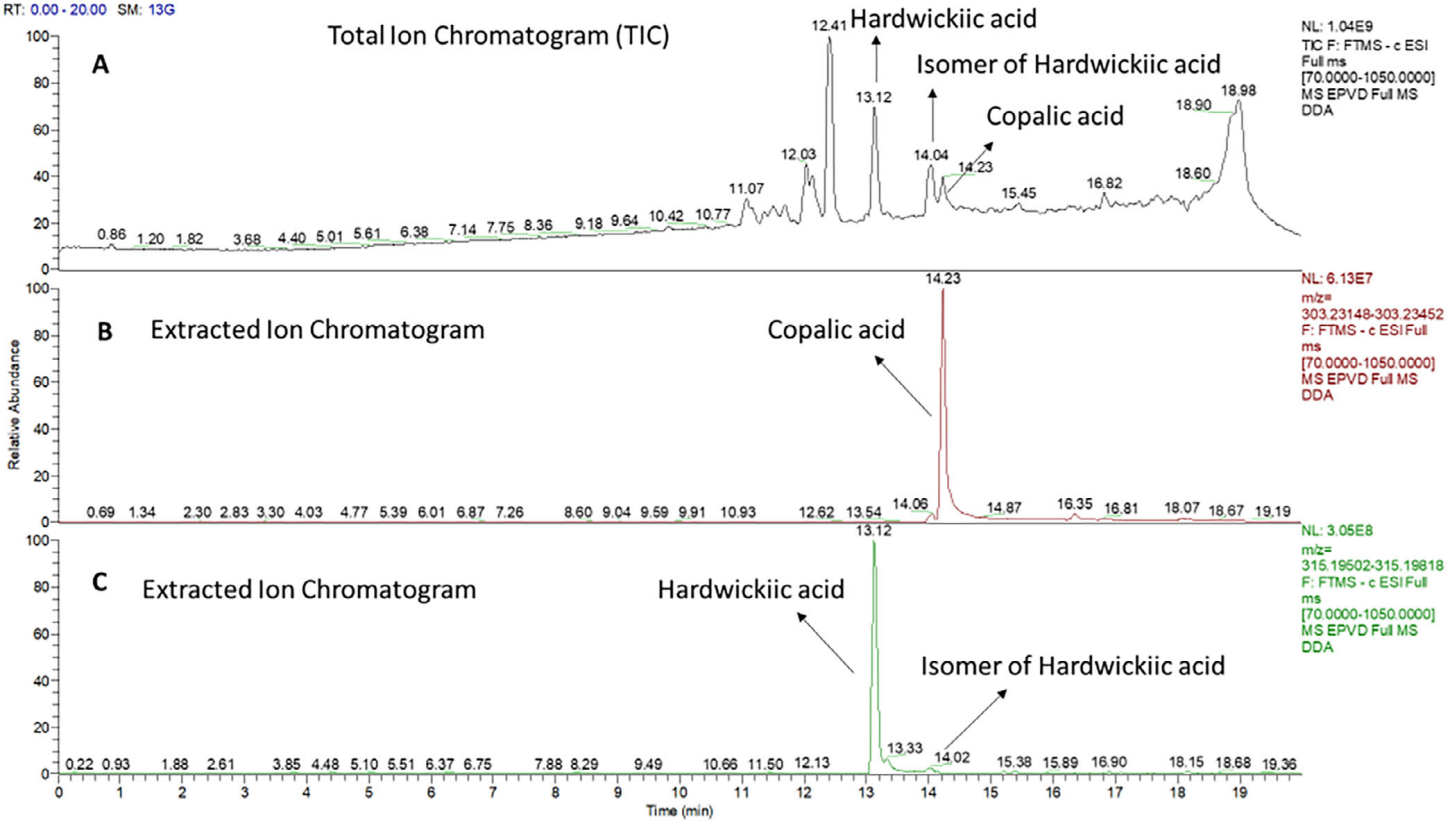
(A) Total ion chromatograms for oleoresin of *Eperua oleifera* Ducke by UHPLC–HRMS. (B) Extracted ion chromatogram of copalic acid (Rt = 14.23 min); (C) extracted ion chromatogram of hardwickiic acid and isomer of hardwickiic acid (Rt = 13.12 and 14.02 min, respectively).

To unequivocally describe the structures of the diterpenic acids, an ion‐change open column chromatography procedure was applied to separate the carboxylic acids from other non‐acid components, so far, alcohols. The acid and non‐acid separation was performed using KOH‐impregnated silica gel prepared in‐house. Subsequent chromatographic column separation of the acidic fraction yielded a purer fraction in mobile phase 8:2 hexane/ethyl acetate, as confirmed by ultra high‐performance liquid chromatographic analysis (UHPTLC), compared with literature and previously isolated standards, showing a single retention factor (Rf) value of 0.54. UHLC–HRMS analysis of this fraction (Figure 2) revealed the presence of three compounds: copalic acid (C_20_H_32_O_2_, ESI–HRMS at *m*/*z* 303.2330 [M−H]^−^) with a retention time (Rt) of 14.23 min; hardwickiic acid (C_20_H_28_O_3_, ESI–HRMS at *m*/*z* 315.1966 [M−H]^−^) with an Rt of 13.12 min; and an isomer of hardwickiic acid with an Rt of 14.02 min, as observed at TIC and EIC.

**FIGURE 3:**
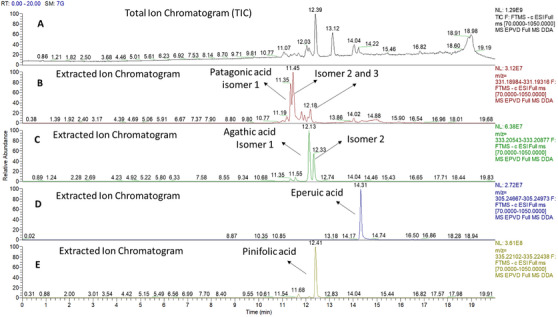
(A) Total ion chromatograms for oleoresin of *Eperua oleifera* Ducke by UHPLC–HRMS. (B) Extracted ion chromatogram of patagonic acid (Rt = 11.35 min), isomer 2 (Rt = 11.45 min), and isomer 3 (Rt = 12.18 min). (C) Extracted ion chromatogram of agathic acid and isomer 2 (Rt = 12.13 and 12.33 min, respectively). (D) Extracted ion chromatogram of eperuic acid (Rt = 14.31 min) and (E) extracted ion chromatogram of pinifolic acid (Rt = 12.41 min).

Patagonic (C_20_H_28_O_4_, HR–ESI–MS at *m*/*z* 331.1915 [M−H]^−^), agathic (C_20_H_30_O_4_, HR–ESI–MS at *m*/*z* 333.2071 [M−H]^−^), eperuic (C_20_H_34_O_2_, HR–ESI–MS at *m*/*z* 305.2482 [M−H]^−^), and pinifolic (C_20_H_32_O_4_, HR–ESI–MS at *m*/*z* 335.2227 [M−H]^−^) acids were also identified, as can be seen in Figure 3.

Copalic and hardwickiic acids had their structures confirmed by NMR experiments, which were inconclusive regarding the isomer. The hybrid quadrupole‐orbitrap mass spectrometer offers a range of scan modes that provide functionality comparable to that of conventional tandem quadrupole mass spectrometers. As examples, the product ion, fragment ion, and data‐dependent neutral loss trigger scan modes are available. Most importantly, the Orbitrap analyzer of the Q Exactive mass spectrometer is a trapping, not a scanning, device [29]. This tool offers greater sensitivity compared to analytical methods commonly used in the analysis of natural products. It will be further applied to *Eperua* oilresins.

**FIGURE 4:**
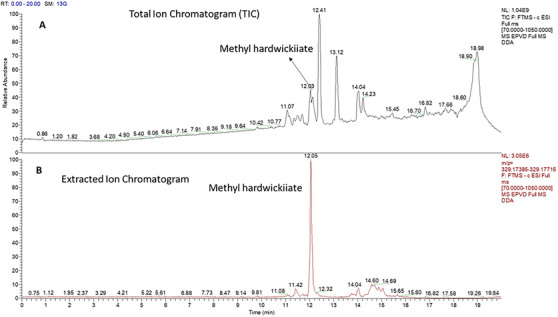
(A) Total ion chromatograms for oleoresin of *Eperua oleifera* by UHPLC–HRMS. (B) Extracted ion chromatogram of methyl hardwickiate (Rt = 12.05 min).

The neutral fraction contained a compound that, after isolation by column chromatography, was identified based on its fragmentation patterns in the mass spectrum and spectral bands in the infrared spectrum. The IR spectrum showed characteristic bands corresponding to the furan ring and the conjugation of the α,β‐unsaturated group with the methyl ester carbonyl at 1710 cm^−1^ [17, 30]. Other bands were observed at 2952, 1758, 1647, 1433, 1249, 939, 754, and 666 cm^−1^ (Figure S2). Full MS analysis in negative mode confirmed the presence of an ion at *m*/*z* 329.1755, with a retention time of 12.03 min (Figure 4). DDA acquisition generated fragment ions at *m*/*z* 285.1863 and *m*/*z* 257.1913. Three replicates of the sample were prepared and injected into the UHPLC–HRMS system. Table S1 presents the area results and the coefficient of variation for the samples injected in triplicate. For all substances, coefficient of variation values below 10% were obtained, including for methyl hardwickiate.

**FIGURE 5:**
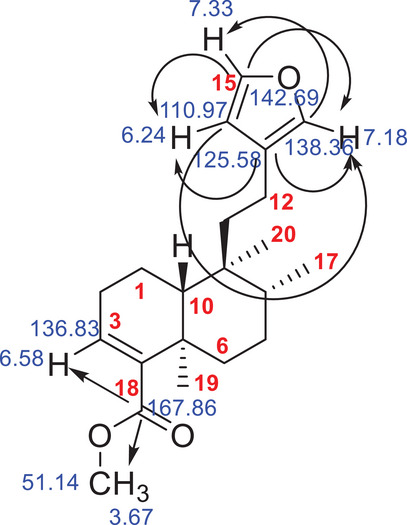
Hardwickiic acid methyl ester chemical structure with some ^1^H and ^13^C NMR and HMBC correlations (arrows).

According to literature data, this corresponds to hardwickiic acid methyl ester (C_21_H_30_O_3_), which was previously isolated as a minor compound from *Echinodorus grandiflora* [31]. The mass error was calculated from the molecular formula, and 0.3 ppm was found. The structure of the methyl ester of hardwickiic acid was also analyzed using 1D and 2D NMR spectroscopy and compared with previously published data [17]. The HMBC spectrum revealed characteristic chemical shift correlations for the hardwickiic diterpene structure (Figure S10), including correlations between C‐13 (*δ* 125.58) and H‐14 (*δ* 6.24) and H‐16 (*δ* 7.18); C‐16 (*δ* 138.2) and H‐15 (δ 7.33); C‐14 (*δ* 110.97) and both H‐15 and H‐16; and C‐15 (*δ* 142.69) with H‐14 and H‐16 (Figure 5). In addition, a correlation was observed between the carbonyl carbon C‐18 (*δ* 167.86) and the methyl protons H_3_‐21 (*δ* 3.67), as well as the methoxy carbon C‐21 (*δ* 51.14), confirming the compound as the methyl ester of hardwickiic acid.

Methyl esters have not been previously reported as natural constituents in oilresins, which may be related to the analytical techniques employed. Esterification is a common practice applied to polar analytes in GC. Carboxylic acids are often observed as their respective methyl esters, overlooking the possibility of naturally occurring methyl esters. To our knowledge, this study is the first of its kind in the literature. To exclude the possibility that the methyl ester was formed as an adduct during the fractionation processes, the oilresin was also directly analyzed by direct insertion on HRMS to confirm that the methyl ester is a natural substance, and the presence of natural methyl hardwickiate was confirmed.

## 3 Conclusions

The oleoresin derived from *E. oleifera* Ducke was analyzed by GC–MS, enabling the detection of its main constituents, which had not been previously reported. Subsequent fractionation by open column chromatography using a KOH‐impregnated silica allowed the separation of acidic and neutral fractions. Further analysis of the acidic fraction led to isolating concentrated portions of hardwickiic and copalic acid, confirmed by NMR and HRMS experiments. In contrast, the neutral fraction contained a natural diterpenic ester—methyl hardwickiic acid—identified through infrared spectroscopy, HRMS, and NMR analyses. This methylated form of hardwickiic acid had not previously been detected in its natural form in oilresins. Direct infusion MS enabled the detection of this ester by providing complementary data to conventional GC–MS methods. This finding opens many studies of methyl esters with natural oilresins.

## 4 Experimental Section

### 4.1 Plant Material

The oilresin from *E. oleifera* Ducke was collected on June 6, 2023, in Manicoré, Amazonas, Brazil. The access was registered under code AAC8B84 in the SISGEN system.

### 4.2 Sample Preparation

The sample preparation procedure consisted of a room temperature derivatization technique in a vial. Ninety microliters of chloroform were added to a vial containing 10 µL of oilresin. After introducing 50 µL of trimethylsulfonium hydroxide (TMSH) reagent, the vial was gently shaken manually until a homogeneous solution was obtained. The total derivatization reaction time was five minutes. Finally, the mixture was brought to a final volume of 1 mL with chloroform up to the meniscus of the vial and injected into the GC [32]. For direct infusion in MS, 1 mg of the sample was dissolved in 1 mL of methanol, GC grade, acquired from Tedia (Fairfield, OH, USA). For UHPLC–HRMS analyses, approximately 1 mg of the sample was weighed in triplicate and quantitatively transferred to 1.00 mL volumetric flasks, which were calibrated and had an expanded measurement uncertainty of less than 0.025 mL. The flasks were vortexed for 1 min and then left to stand for 20 min before being brought to volume. The samples were subsequently transferred to vials for analysis. All stock solutions were stored at −30°C. All volumetric materials used are periodically calibrated by LAB CAL, a laboratory accredited by the Brazilian Calibration Network (CAL 0328 INMETRO), part of LBCD.

### 4.3 Phytochemical Analysis GC–MS Analysis of Oilresin From *E. oleifera* Ducke

The oilresin of *E. oleifera* was analyzed using GC–MS on a Shimadzu QP2020 NX system (Shimadzu Corporation, Kyoto, Japan) equipped with an AOC‐20i autosampler and an SH‐RTX‐5 MS column (Shimadzu) with a stationary phase of 5% phenyl and 95% dimethylpolysiloxane (30 m × 0.25 mm × 0.25 µm). Helium was used as the carrier gas at a flow rate of 1.35 mL/min in constant flow mode. A volume of 1 µL of esterified oilresin was injected in split mode (1:10). The injection port was set to 250°C, as well as the transfer line. The oven temperature was initially set at 180°C for 2 min, then increased to 290°C at a rate of 2°C/min, with a final isothermal hold for 15 min. The mass analyzer operated in total ion scanning mode (full scan) with a *m*/*z* 30–500 range. The transfer line and the ionization source temperatures were set at 300°C and 320°C, respectively. An energy of 70 eV was used for electron ionization. The mass spectra were interpreted using the reference library of the NIST (USA), along with literature data on previously identified diterpenes. The percentage composition of the constituents was determined based on peak area.

### 4.4 KOH‐Impregnated Column Chromatography

A fractionation process was performed using ion‐exchange column chromatography with silica impregnated with KOH to separate the acidic (polar) fraction from the neutral (non‐polar) fraction. After adding the modified silica and the crude oilresin of *E. oleifera*, the column was eluted with dichloromethane to obtain the non‐acidic components, separating them from the diterpenic carboxylic acids. The diterpenic acids remained retained by the KOH‐impregnated silica during the dichloromethane elution. Subsequently, methanol was used to elute diterpenic acids as potassium salts. The methanolic fraction was concentrated under low pressure and immediately acidified with HCl to a pH of 4–5. Dichloromethane was added, and the diterpenic acids were recovered from the organic phase (dichloromethane) in a separation funnel. Both the acidic and neutral fractions were evaporated using a rotary evaporator and then stored at a low temperature until further isolation [12, 17].

### 4.5 Column Chromatography

A chromatographic column was prepared using silica gel (70–230 mesh) as the stationary phase for the isolation and identification of compounds. A quantity of silica was weighed in a 1:10 ratio relative to the amount of sample used for each fractionation. The column was packed by adding silica that had been pre‐soaked in the designated mobile phase to a glass column. For the acidic fraction, the mobile phase started with an 8:2 ratio of hexane/acetate, gradually increasing in polarity as fractions were collected at each gradient, until reaching a final mobile phase ratio of 1:1. For the neutral fraction, an isocratic mobile phase of 9:1 (hexane/acetate) was used throughout the entire column. The solvent was evaporated using a rotary evaporator, and the obtained fractions were stored at a low temperature until further analysis.

### 4.6 Direct Infusion in MS

Mass spectrometer Q Exactive Orbitrap operates in a positive and negative ESI mode and is calibrated daily with a manufacturer's calibration solution (Thermo Fisher Scientific, Bremen, Germany). ESI parameters were further optimized with the final setup: spray voltage of 2.9 kV, S‐lens voltage of 80 V, the capillary temperature of 380°C, auxiliary gas heater temperature of 350°C, nitrogen sheath, auxiliary, and sweep gas were set at 30, 10, and 1 arbitrary units, respectively. Full‐scan data were acquired in a range of *m*/*z* 100–1000 at a resolution of 140 000 full widths at half maximum (FWHM), automatic gain control (AGC) of 1 × 10^6^, and maximum injection time (IT) of 100 ms.

Data was acquired in a Full MS/dd‐MS^2^ mode (without HCD fragmentation), followed by data‐dependent (dd) scans with fragmentation energy applied. Ions of the second scan event enter the HCD collision cell; ions of the first do not.

The analysis used high‐resolution mass spectrometry (HRMS) by direct infusion. The sample was dissolved in methanol and infused into the ESI ionization source in positive and negative modes. Thermo Scientific TraceFinder 4.1 software (Thermo Fisher Scientific, Austin, TX, USA), with a ±5 ppm mass tolerance.

### 4.7 UHPLC–HRMS Parameters

A Dionex Ultimate 3000 UHPLC system coupled to a QExactive Plus hybrid quadrupole Orbitrap mass spectrometer (Thermo Fisher Scientific) equipped with an electrospray ionization (ESI) source was used. Separation was performed in a reversed‐phase column (Kinetex 2.6 µm PS C18, 100 Å, 100 mm × 2.1 mm; 2.6 µm) at 40°C, with a constant flow rate of 300 µL/min and an injection volume of 5 µL. A gradient chromatographic run started at 5% of mobile phase B (methanol with 0.1% formic acid) and 95% of mobile phase A (water with 5 mM ammonium formate and 0.1% formic acid). Mobile phase B increased to 10% at 1.0 min, 25% at 2 min, and 90% at 10 min. After reaching 100% of B at 14 min and maintaining this ratio until 16 min, the initial chromatographic condition was restored from 16.1 to 20.0 min.

The LC effluent was pumped to the mass spectrometer operating in a positive ESI mode, calibrated daily with a manufacturer's calibration solution (Thermo Fisher Scientific). ESI parameters were further optimized with the final setup: spray voltage of 2.9 kV, S‐lens voltage of 80 V, the capillary temperature of 380°C, auxiliary gas heater temperature of 350°C, nitrogen sheath, auxiliary, and sweep gas were set at 30, 10, and 1 arbitrary unit, respectively. Full‐scan data were acquired in a range of *m*/*z* 70–1050 at a resolution of 70,000 FWHM, AGC of 1 × 10^6^, and maximum IT of 100 ms.

Data were acquired in a Full MS/dd‐MS2 mode (without HCD fragmentation), followed by data‐dependent (dd) scans with fragmentation energy applied. Ions of the second scan event enter the HCD collision cell; ions of the first do not.

The analysis used HRMS by direct infusion. The sample was dissolved in methanol and infused into the ESI ionization source in positive and negative modes. Thermo Scientific TraceFinder 4.1 software (Thermo Fisher Scientific), with a ±5 ppm mass tolerance.

### 4.8 Precision Evaluation

The precision was evaluated through % RSD, calculated for each substance.

### 4.9 Fourier Transform Infrared Spectroscopy Analysis

The spectra of the isolated compound were obtained using Fourier transform infrared (FTIR) spectroscopy on a Hewlett‐Packard FTIR instrument. The infrared absorbance data of a minimal amount of the dried oily compound were recorded in the wavenumber range of 4000–600 cm^−1^.

### 4.10 NMR

NMR experiments of the isolated diterpenic acids and the methyl ester compounds were performed on a 600 MHz Agilent spectrometer operating at 599.87 MHz for ^1^H and 150.85 MHz for ^13^C, at 298 K, using a 5 mm NMR tube in CDCl_3_. Spectra were taken using the standard pulse sequence. The ^1^H NMR spectrum was acquired with a single 45° radiofrequency excitation pulse, a spectral width of 5.38 kHz, an acquisition time of 1.52 s, 8 k data points, and 16 scans. The ^13^C NMR spectrum was acquired with a single 45° radiofrequency pulse, a spectral width of 30.5 kHz, an acquisition time of 1.05 s, a relaxation delay of 2 s, and 32 k data points. The DEPT 135 pulse sequence was performed using a 30.5 kHz spectral width. COSY spectrum was performed using the same spectral width as the ^1^H spectrum, with 1024 (*t*_2_) × 512 (*t*_1_) complex data points. NOESY spectra were obtained applying a 300 ms mixing time. HSQC experiment with adiabatic carbon pulses and gradient pulses was acquired with 1024 (*t*_2_) × 256 (*t*_1_) complex data points, and 16 scans were employed. HMBC was acquired with 1024 (*t*_2_) × 256 (*t*_1_) complex data points, and 32 scans. All spectra were processed and analyzed using MestreNova software.

## Author Contributions

All authors contributed to the conception and design of the study. Rayssa Ribeiro: GC–MS injection, analysis and interpretation of the results. HRMS experiments: Valdir F. Veiga‐Junior and Monica C. Padilha. NMR experiments: Valdir F. Veiga‐Junior, Alvicler Magalhães and Fernando Hallwass. All authors reviewed, commented on, and approved the final version of the manuscript.

## Conflicts of Interest

Authors declare no conflict of interest.

## Supporting information




**Supporting File 1**: cbdv70536‐sup‐0001‐SuppMat.docx

## Data Availability

The data that support the findings of this study are available from the corresponding author upon reasonable request.
