# Supplementary material for: Eperua oleifera Ducke (Fabaceae) Oilresin Chemical Composition and the Isolation of a Natural Diterpenic Acid Methyl Ester
Source: Chem Biodivers. 2025 Sep 25;22(12):e01730. doi: 10.1002/cbdv.202501730 (PMC12716019; doi:10.1002/cbdv.202501730)
Supplement: Supplementary file 1 — Supporting File 1: cbdv70536‐sup‐0001‐SuppMat.docx [file CBDV-22-e01730-s001.docx]

**Supporting Information**


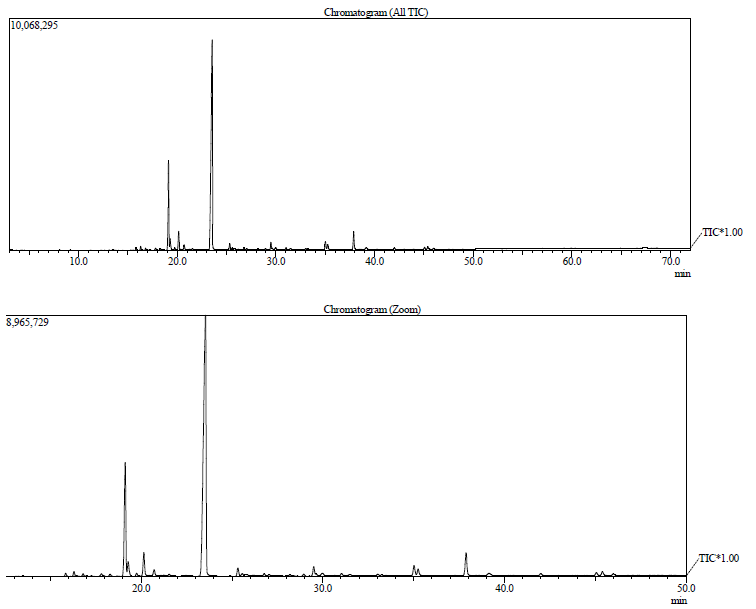


**a**

**b**

Figure S1. Total ion chromatograms (TIC) for oleoresin of *Eperua oleifera* Ducke by GC-MS (**a**). Expanded total ion chromatograms (TIC) (**b**).


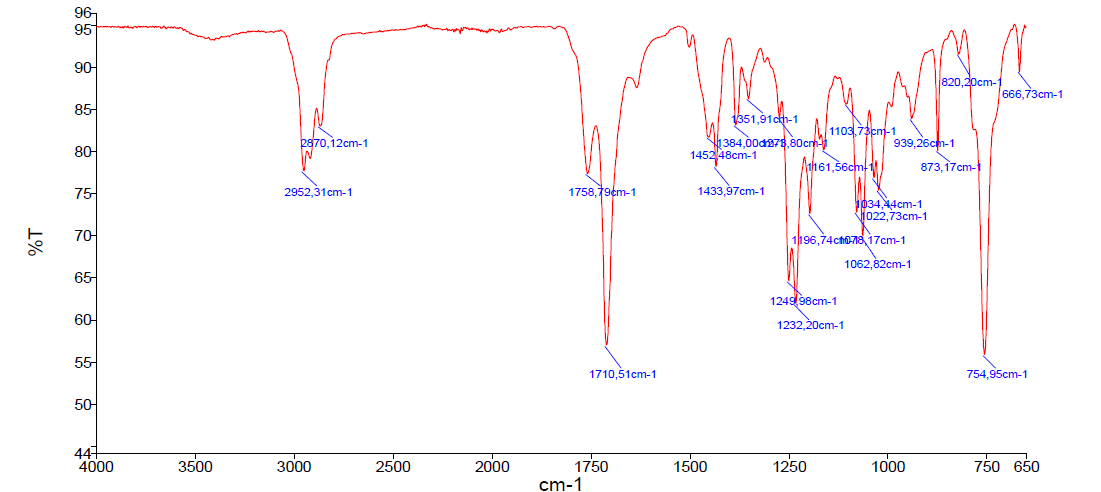


Figure S2. Infrared spectrum of the isolated hardwickiic methyl ester.


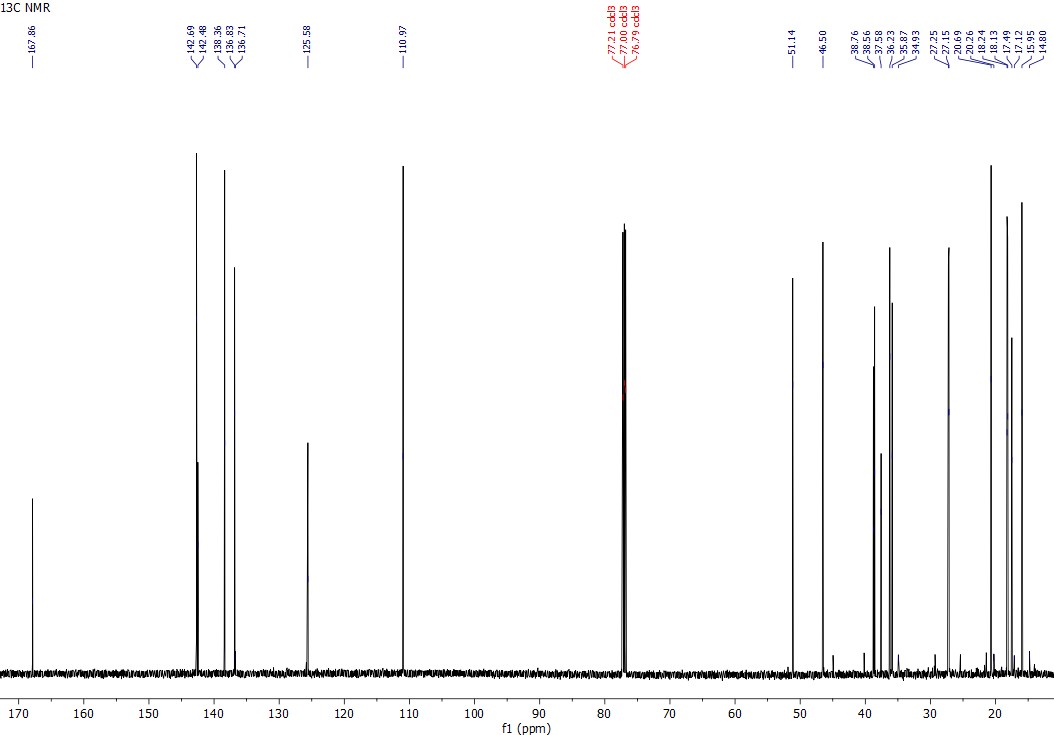


Figure S3. ^13^C NMR spectrum of the isolated hardwickiic methyl ester.


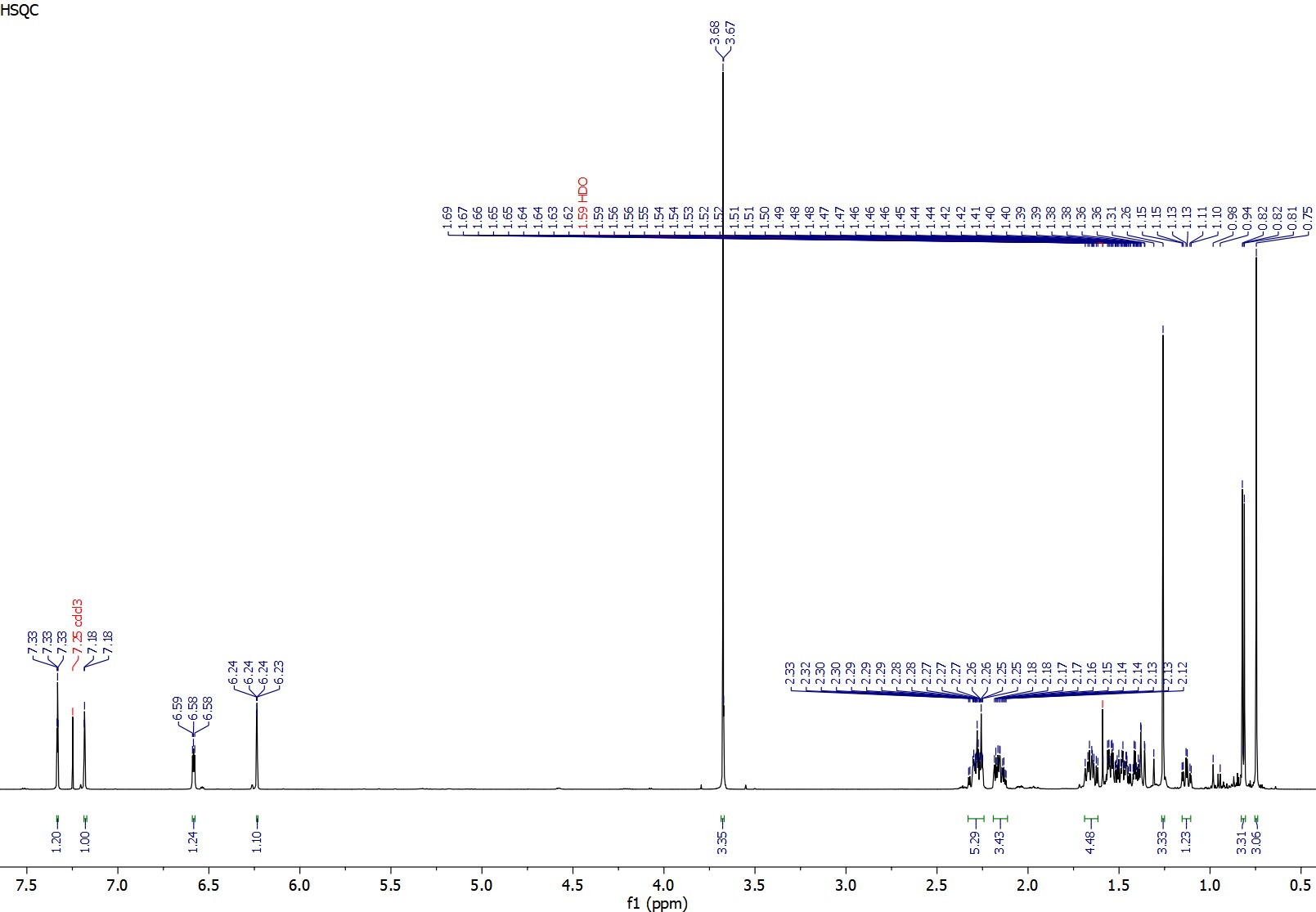


Figure S4. ^1^H NMR spectrum of the isolated hardwickiic methyl ester.


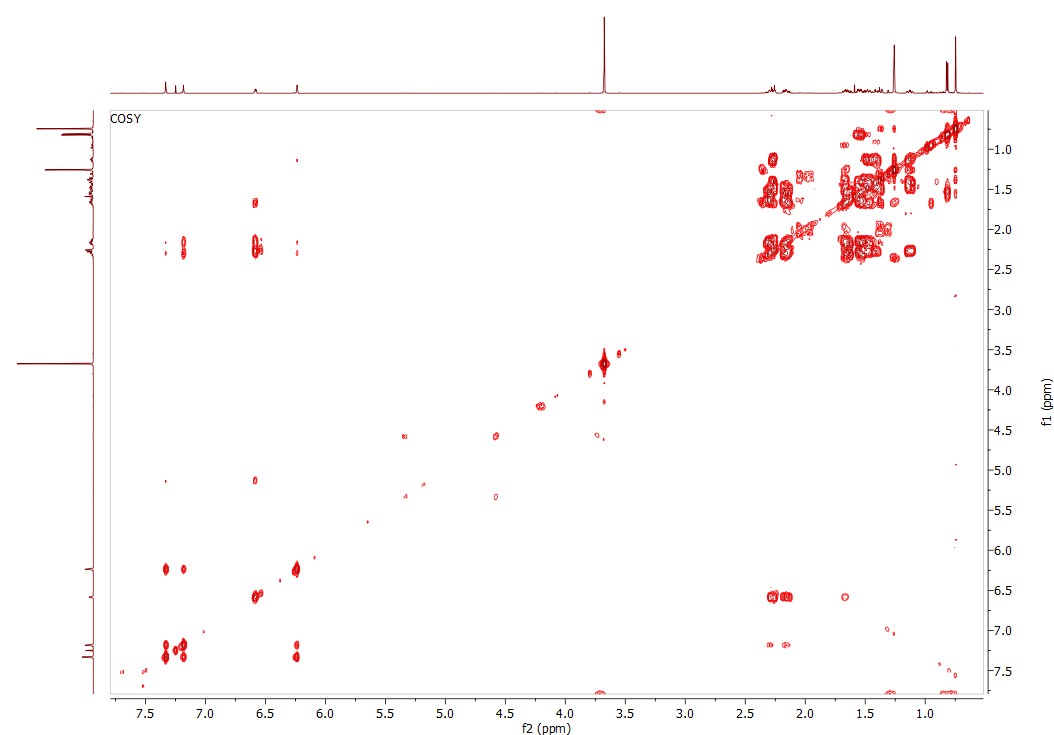


Figure S5. COSY spectrum of the isolated hardwickiic methyl ester.


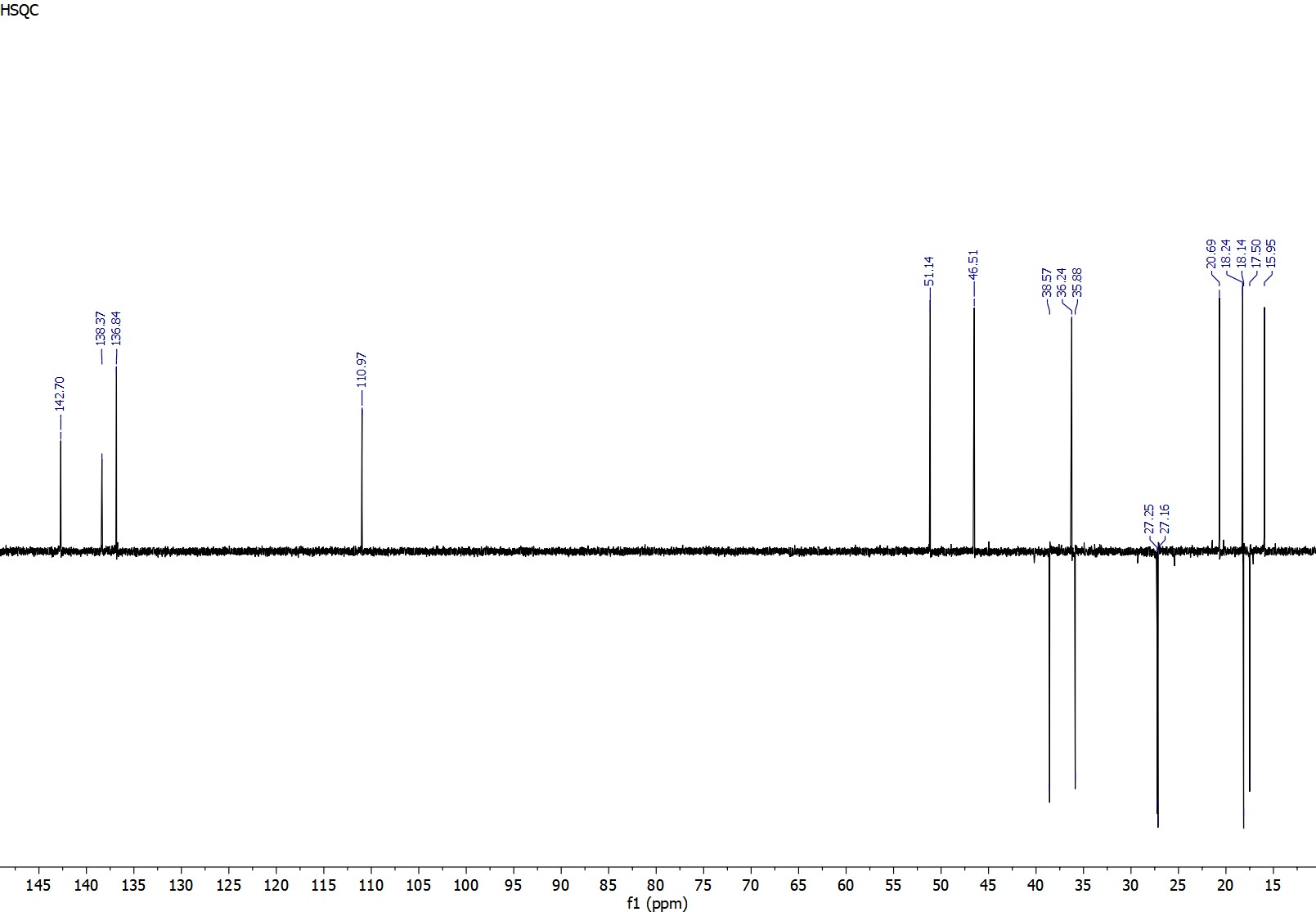


Figure S6. DEPT spectrum of the isolated hardwickiic methyl ester.


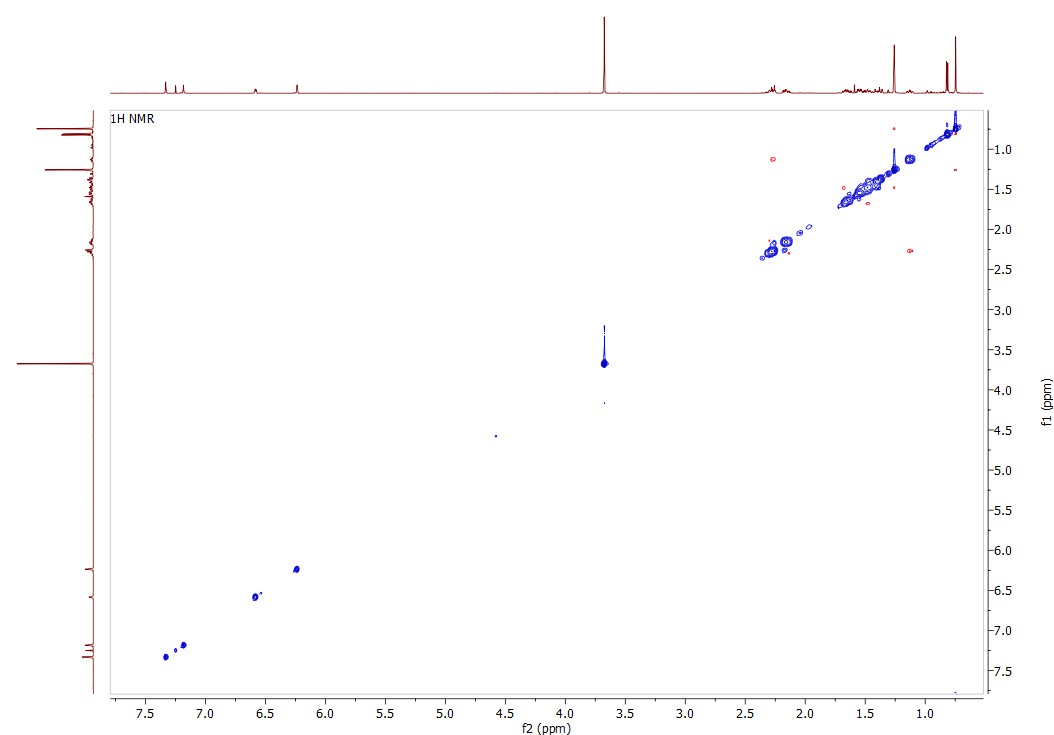


Figure S7. NOESY spectrum of the isolated hardwickiic methyl ester.


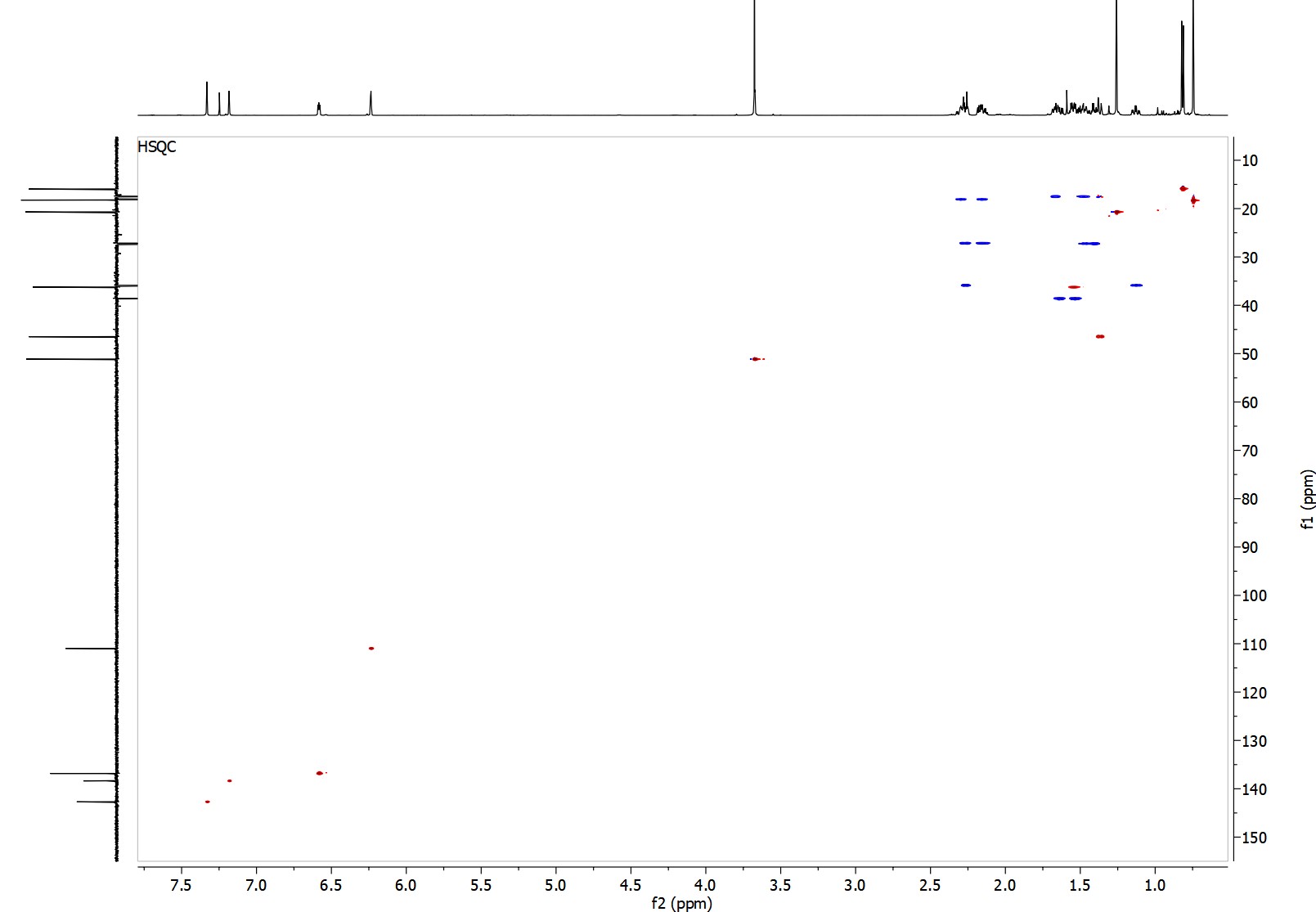


Figure S8. HSQC spectrum of the isolated hardwickiic acid methyl ester.


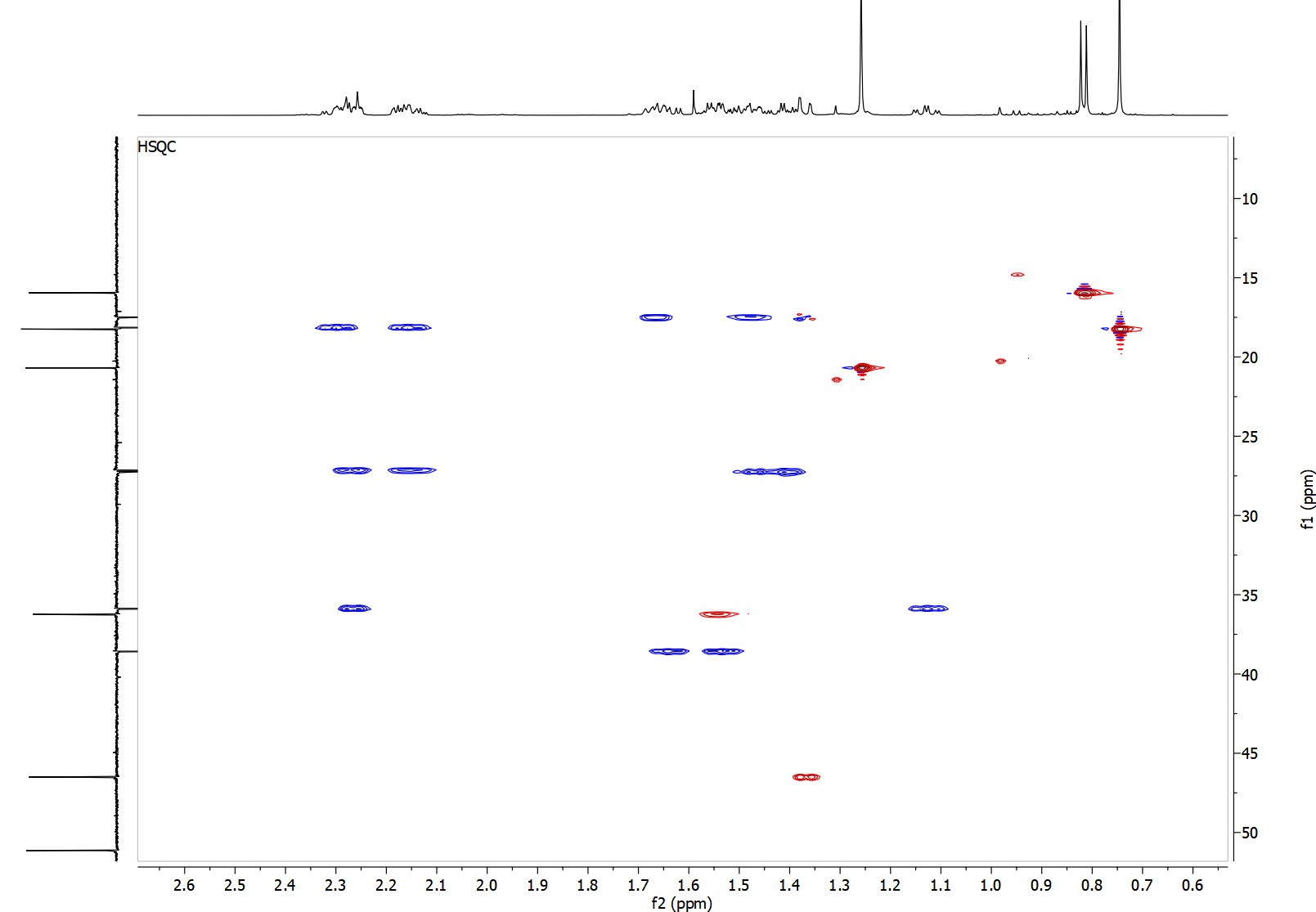


Figure S9. Extended HSQC spectrum of the isolated hardwickiic acid methyl ester: CH2 in blue and CH/CH3 in red.


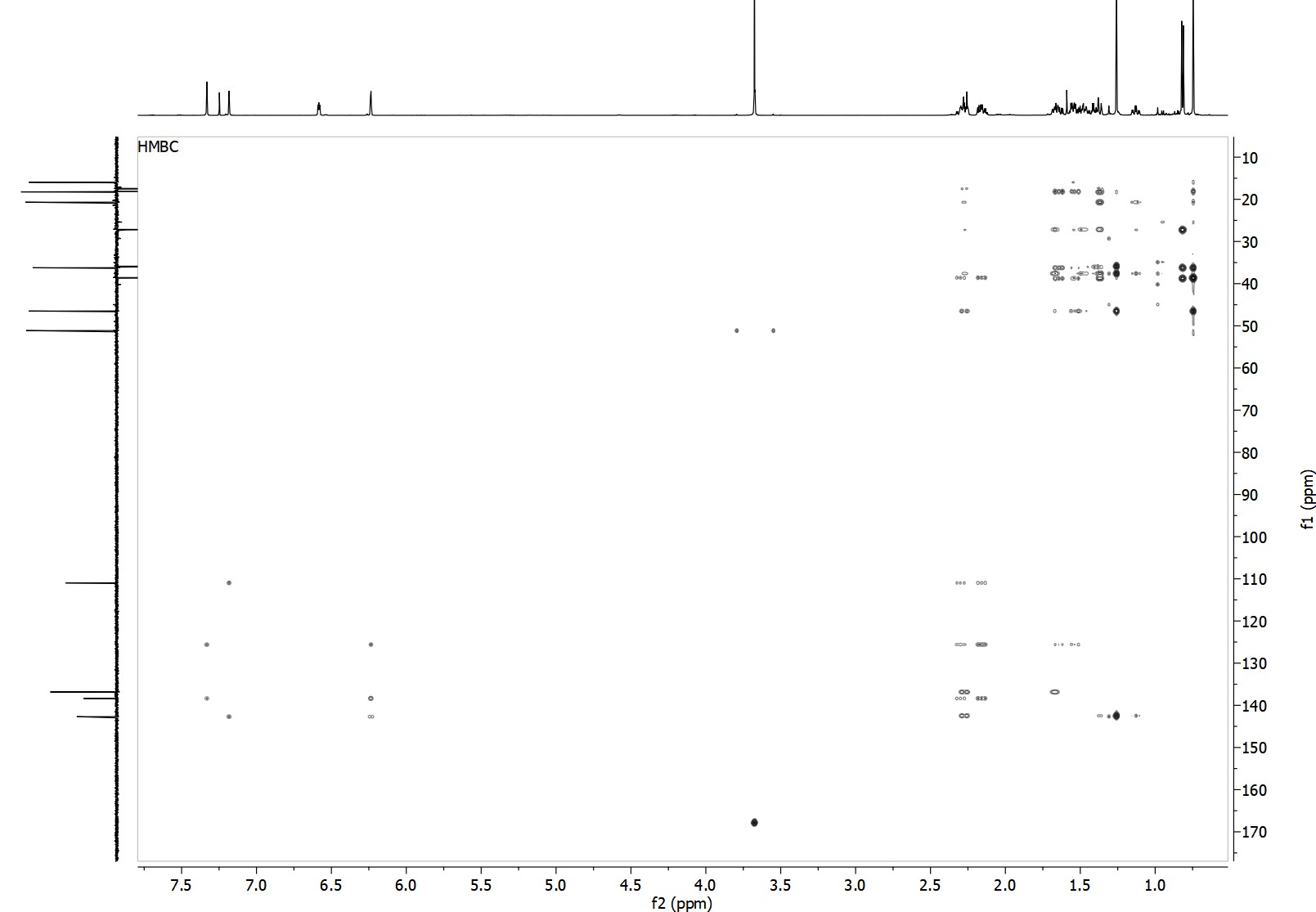


Figure S10. HMBC spectrum of the isolated hardwickiic acid methyl ester


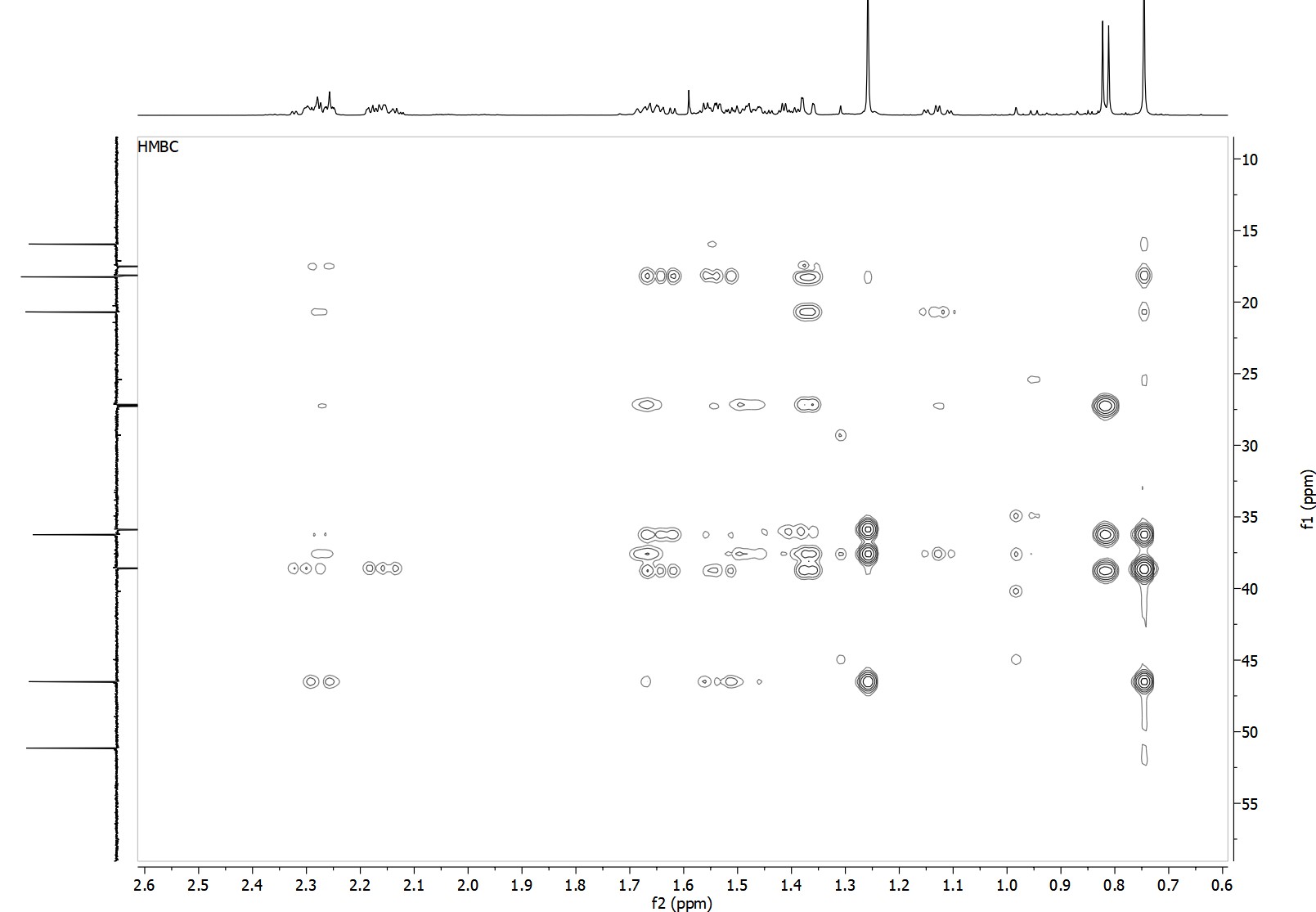


Figure S11. Extended HMBC spectrum of the isolated hardwickiic acid methyl ester.

Table S1. Area of the diterpene acids and their methyl hardwickiate UHPLC. Oilresin dissolved in methanol.

| **Substances** | **Retention time (min)** | **Samples** | | | **CV%** |
| --- | --- | --- | --- | --- | --- |
|  |  | **Replicate 1** | **Replicate 2** | **Replicate 3** |  |
| Hardwickiic acid | 13.12 | 874648197 | 899377758 | 754678649 | 9,2 |
| Isomer of hardwickiic acid | 14.02 | 45305784 | 44770269 | 41835164 | 4,2 |
| Copalic acid | 14.23 | 207561117 | 213274955 | 189753257 | 6,0 |
| Patagonic acid isomer 1 | 11.35 | 58904109 | 59661382 | 56324552 | 3,0 |
| Patagonic acid isomer 2 | 11.45 | 117340195 | 112222247 | 106958176 | 4,6 |
| Agathic acid isomer 1 | 12.13 | 206574788 | 191279891 | 186581543 | 5,4 |
| Agathic acid isomer 2 | 12.33 | 92329156 | 109410764 | 99537176 | 8,5 |
| Eperuic acid | 14.31 | 60524874 | 64801787 | 58908448 | 5,0 |
| Pinifolic acid | 12.41 | 1101207972 | 1077632249 | 1062126111 | 1,8 |
| Methyl hardwickiiate | 12.05 | 17150755 | 16065843 | 15265808 | 5,9 |
